# Proximal metatarsal osteotomy for hallux valgus: an audit of radiologic outcome after single screw fixation and full postoperative weightbearing

**DOI:** 10.1186/1757-1146-6-22

**Published:** 2013-05-31

**Authors:** Falk Mittag, Ulf Leichtle, Christoph Meisner, Ingmar Ipach, Nikolaus Wülker, Markus Wünschel

**Affiliations:** 1 Department of Orthopaedics, University Hospital Tuebingen, Hoppe-Seyler-Strasse 3, 72076 Tuebingen Germany; 2 Department of Medical Biometry, University Tuebingen, Westbahnhofstrasse 55, 72070 Tuebingen, Germany

**Keywords:** Hallux valgus, Proximal metatarsal osteotomy, Single screw fixation, Full weightbearing

## Abstract

**Background:**

Proximal metatarsal osteotomy combined with a distal soft-tissue procedure is a common treatment for moderate to severe hallux valgus. Secure stabilisation of the metatarsal osteotomy is necessary to avoid complications such as delayed union, nonunion or malunion as well as loss of correction. The aim of this study was to report our results using a single screw for stabilisation of the osteotomy.

**Methods:**

We retrospectively reviewed 151 patients with severe hallux valgus who were treated by the above mentioned way with full postoperative weightbearing in a stiff soled shoe. Mean age of patients at time of surgery was 54 years, 19 patients were male and 132 female. Assessment of clinical and radiographic results was performed after 2 days and 6 weeks. Results were also correlated to the experience of the performing surgeon.

**Results:**

Mean preoperative HVA (hallux valgus angle) was 36.4 degrees, and then 3.5 degrees 2 days and 13.4 degrees 6 weeks after the procedure (*p* < 0.001). Mean preoperative IMA (intermetarsal angle) was 16.8 degrees, and then 6.4 degrees after 2 days and 9.8 degrees after 6 weeks (*p* < 0.001). Mean preoperative first metatarsal length of 56.4 mm decreased to 53.6 mm after 6 weeks. Possible non-union of the osteotomy was observed in 4 patients (2.6%) after 6 weeks. Performing residents (n = 40) operated in 65 minutes and attending surgeons (n = 111) in 45 minutes, with no significant differences in radiographic measurements between both groups.

**Conclusions:**

Single screw stabilisation of proximal chevron osteotomy is a reliable method for treating severe hallux valgus deformities with satisfactory results.

## Introduction

Hallux valgus and lesser toe deformities are common foot deformities presenting to foot and ankle surgeons. For moderate and severe hallux valgus deformities, with intermetatarsal angles (IMA) exceeding 15 degrees, proximal osteotomies of the first metatarsal, combined with a distal soft-tissue procedure, became a common treatment after being introduced by Mann [1,2].

During the past decades, multiple modified procedures and fixation techniques have been described. The most common types of proximal osteotomies are crescentic, chevron, oblique and lateral closing or medial opening wedge techniques. Over the past 10 years, some surgeons have increasingly used locking plates instead of a single screw for stabilisation of the osteotomy with the assumption of better postoperative stability and preserved length of the first metatarsal. In addition, fixation with a plate has been described as technically less challenging [3-8]. However, we prefer a proximal open-wedge chevron-like osteotomy combined with medial bone impaction and single screw stabilisation [3].

Therefore, the aim of this project was to report our experience with special regard to early postoperative loss of correction, change of metatarsal length and non-union with this procedure.

## Methods

From 2006 to 2011, 151 consecutive patients with moderate to severe hallux valgus deformity and intermetatarsal angles greater 15 degrees were treated with a distal soft tissue procedure, removal of medial eminence of the first metatarsal head and proximal chevron osteotomy at our institution. After the procedure patients were mobilized with full weightbearing wearing a shoe with a stiff sole for six weeks after surgery. The mean age of patients at time of surgery was 54 years (range 15–73), 19 were male and 132 were female. All patients participating in this study gave informed consent.

Retrospectively, preoperative and postoperative (1–2 days and 6 weeks after surgery) radiographs were examined. No additional study related procedures or examinations were performed. The IMA, hallux valgus angle (HVA) and length of first metatarsal were measured on weightbearing anteroposterior radiographs of the foot. To determine the amount of first metatarsal protrusion, length of the first and second metatarsal was calculated using the method described by Hardy and Clapham [9]. A positive sign indicates that the first metatarsal is longer than the second and a negative sign that the second is longer than the first.

Results (IMA, HVA, MT1-length, operating time) of the performing surgeon (attending vs. residents in the 2nd to 6th year of residency) were corroborated whereby the performing resident was always assisted by an attending physician.

### Statistical analysis

For statistical analysis SAS version 9.1.3 (SAS Institute, Cary/NC, USA) biostatistical software was used in cooperation with the Department of Medical Biometry Tübingen, Germany. Quantitative variables with normal distribution were expressed as mean ± standard deviation. Student’s *t*-test was used to test the hypotheses regarding differences between the variables (IMA, HVA, length of first metatarsal, MT-Index) pre- and postoperatively: for HVA a change of 10 degrees, for IMA 5 degrees and for length of first metatarsal and MT-Index a change of 3 mm were considered to be within normal range. Corresponding 99.6% confidence intervals (CI) were calculated to adjust for a significance level of 0.05.

### Operative technique

The procedure was performed under general anesthesia or using sciatic nerve block. An Esmarch bandage was used to obtain a bloodless field. The distal soft tissue procedure was performed through a dorsal incision in the first webspace including tenotomy of the adductor hallucis muscle, division of the deep intermetatarsal ligament and lateral capsule incision of the first metatarsophalangeal joint.

A second midline incision was made over the medial aspect of the first metatarsophalangeal joint and extended proximally along the shaft to the tarsometatarsal joint. Alternatively, two separate incisions were used to expose the medial eminence and proximal metatarsal shaft. After opening the capsule with an inverted L-shaped incision the medial eminence was excised and utilized for autologous bone graft in the proximal osteotomy.

**Figure 1:**
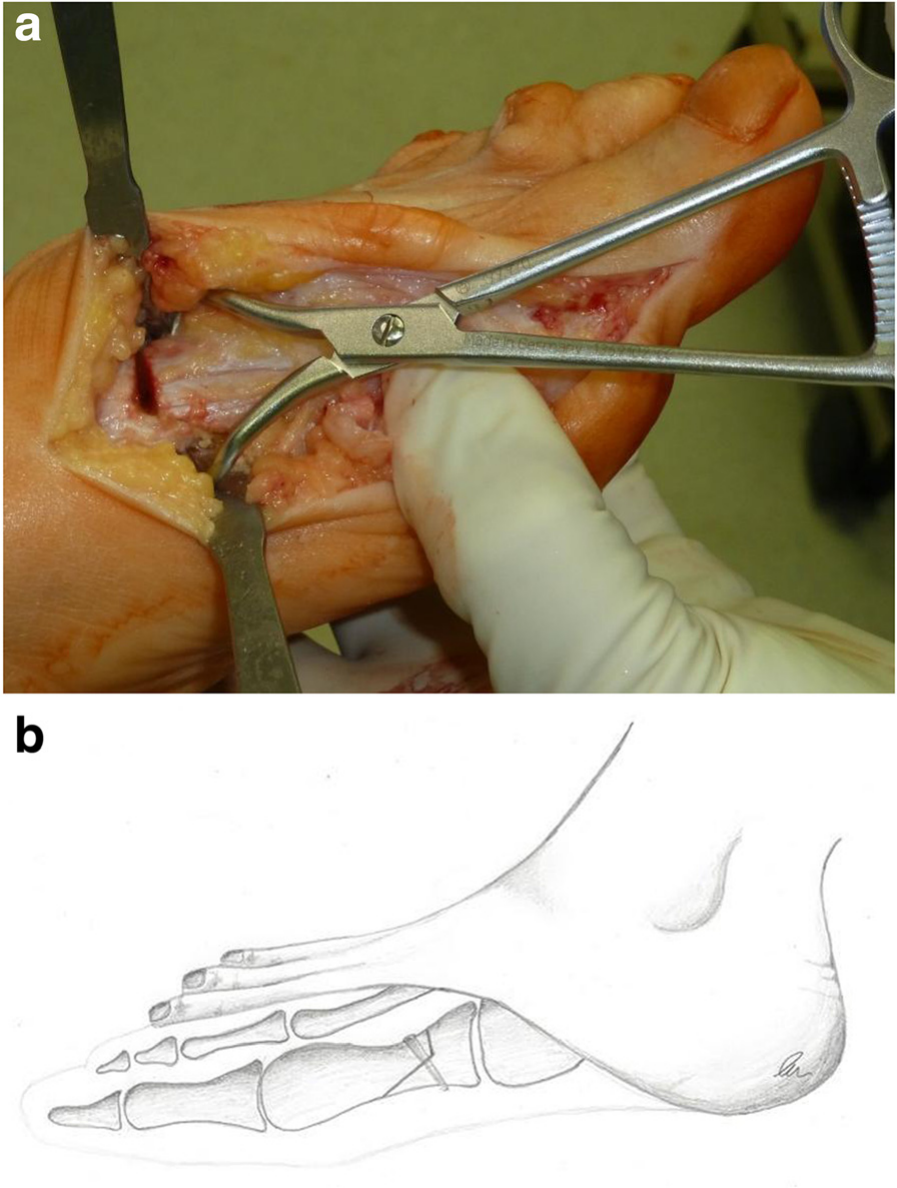
**Proximal metatarsal osteotomy. a**. Intraoperative picture of the proximal chevron-like metatarsal osteotomy 1 cm distal to the tarsometatarsal joint. **b**. Detail drawing of the surgical technique demonstrating the bone cut and and possible screw placement.

**Figure 2:**
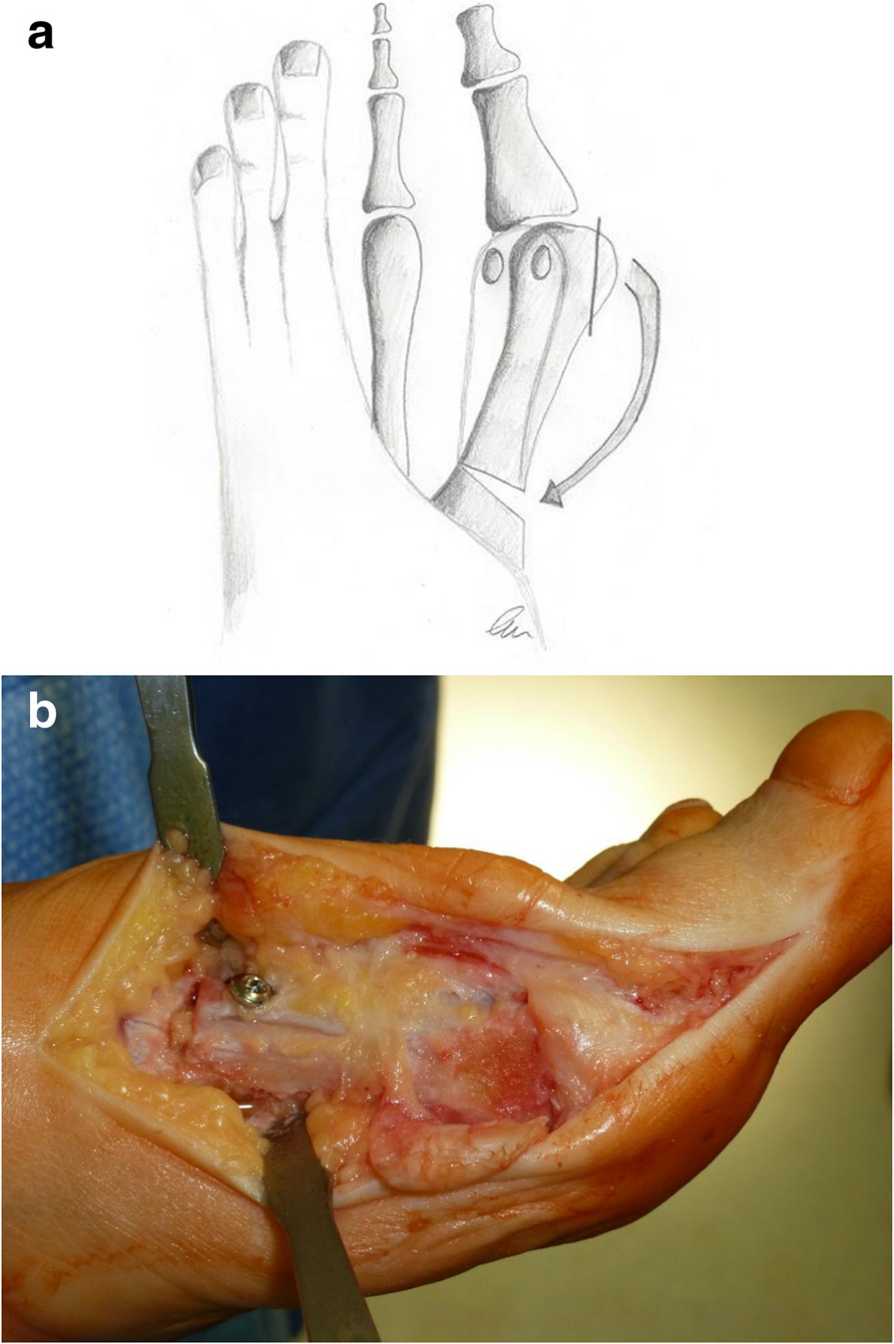
**Correction of the first metatarsal angle and stabilisation of the osteotomy. a**. Schematic diagram showing the correction of the first intermetatarsal angle. The open-wedge osteotomy is additionally stabilized by cortical bone (arrow, see also Figure 2b). **b**. Stabilisation of the osteotomy with a partially threaded cancellous bone screw. The entrance hole was countersunk. Wedge-shaped autologous bone obtained from the medial eminence of the metatarsal head was inserted medially.

**Figure 3:**
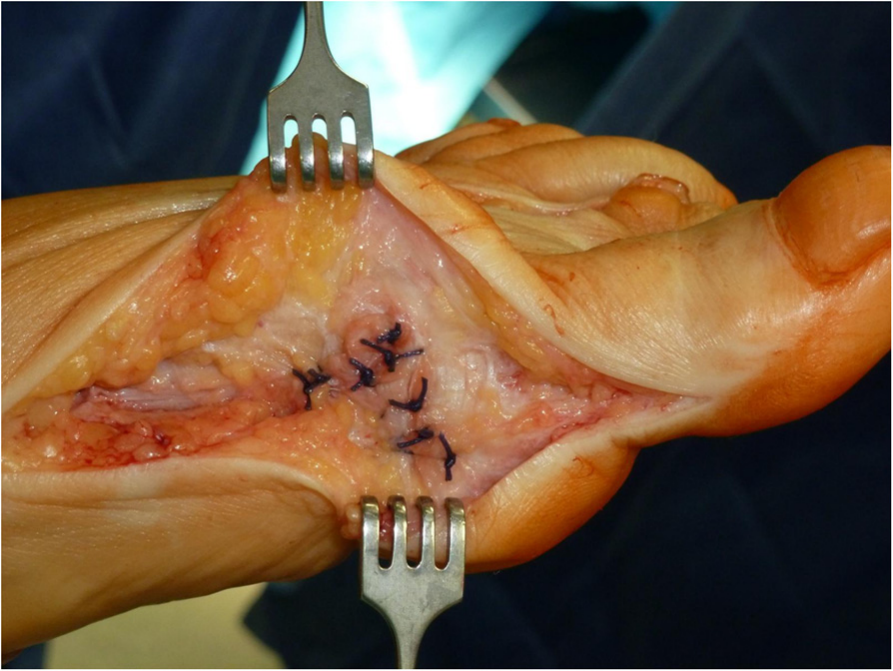
Medial capsulorrhaphy with absorbable sutures.

A chevron-type open wedge osteotomy of the proximal metatarsal was performed with a small oscillating saw approximately 1 cm distal to the tarsometatarsal joint (Figure 1a,b). The proximal fragment was pulled medially while moving the distal fragment laterally. Previously obtained wedge-shaped autologous bone was inserted medially (Figure 2a). The osteotomy was stabilized using a single cancellous bone screw (partially threaded) in a distal-dorsal to proximal-plantar direction (Figure 2b). To reduce the prominence of the head of the screw the entrance hole was countersunk. Finally, the redundant medial capsule was reduced by excision of a capsule-strip and then closed with absorbable sutures (Figure 3).

**Figure 4:**
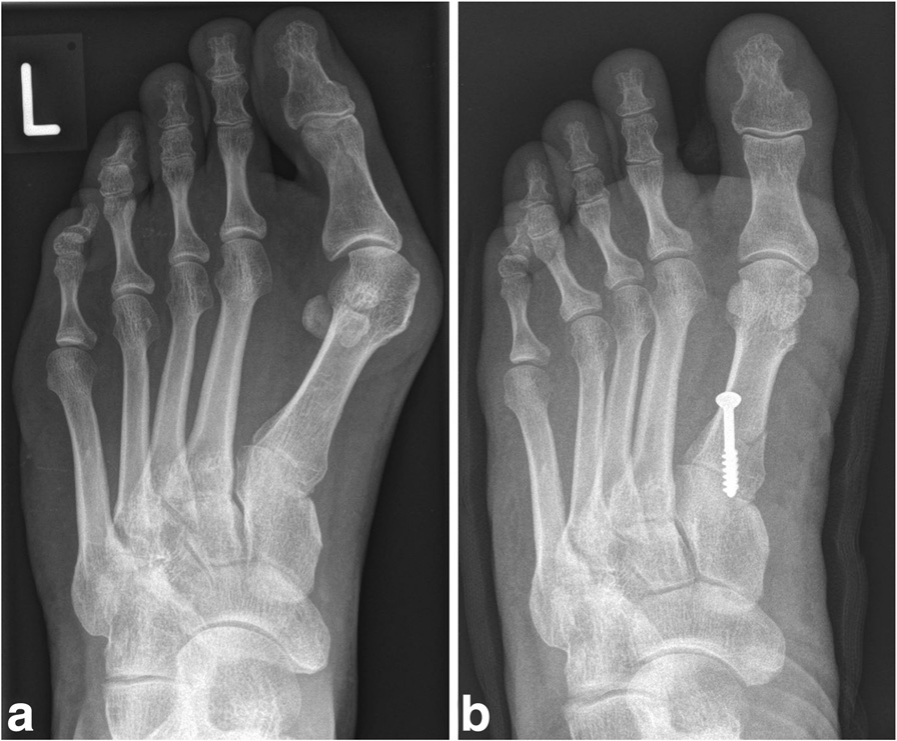
**Pre- and postoperative radiographs.** Weightbearing AP radiographs of the same patient presented in Figures 1, 2, 3: preoperatively (**a**) and two days postoperatively (**b**) showing a well aligned first toe without shortening of the first metatarsal.

To secure correct hallux position a dressing bandage was applied at the time of surgery and continued for the first 6 weeks postoperatively. Immediately after surgery, all patients were allowed full weightbearing in a stiff-soled shoe which was discontinued after clinical and radiological evaluation after 6 weeks (Figure 4).

## Results

Pre- and postoperative measurements are presented in Table 1. Overall the patients had a mean preoperative HVA of 36.4 (range, 17 to 53) degrees. The mean HVA had been significantly reduced to 3.5 (range, -13 to 27) degrees (*p* < 0.001) at 2 days postoperative. Six weeks after surgery, the mean HVA was still significantly reduced at 13.4 (range, -5 to 48) degrees (*p* < 0.001). The overall mean IMA preoperatively was 16.8 (range, 14 to 25) degrees, and then 6.4 (range, 0 to 14) degrees after 2 days (*p* < 0.001) and 9.8 (range, 0 to 24) degrees after 6 weeks (*p* < 0.001).

**Table 1:** **Radiographic measurements (degrees/mm ± standard deviation**)

	**preop.**	**2 days postop.**	**6 weeks postop.**	**Change** **(preop. vs. 6 weeks postop.)**
HVA, degrees	36.4 ± 7.0	3.5 ± 7.9	13.4 ± 10.8	−23 (*p* < 0.001)
IMA, degrees	16.8 ± 3.1	6.4 ± 2.9	9.8 ± 4.5	−7 (*p* < 0.001)
First MT length, mm	56.4 ± 5.0	57.5 ± 5.5	53.6 ± 5.6	−2.8 (*p* = 0.52)
First MT protrusion, mm	4.7 ± 3.2	2.9 ± 3.4	2.5 ± 3.5	−2.2 (*p* = 0.67)

Notes: *HVA* hallux valgus angle, *IMA* intermetatarsal angle, *MT* metatarsal.

Mean preoperative first metatarsal length was 56.4 mm, and then 57.5 mm after 2 days and 53.6 mm after 6 weeks, which was not statistically significant. We also found no statistically significant change for pre- and postoperative measurements for first metatarsal protrusion.

The mean overall operating time was 50 minutes. Performing residents (n=40) operated in 65 minutes, while performing attending surgeons (n=111) operated in 45 minutes. We found no significant differences between residents and attending physicians concerning change of HVA, IMA or MT I-length during follow up.

At the 6 week follow-up, 4 patients (2.6%) presented with a possible non-union of the metatarsal osteotomy that required further immobilization of the foot (2 patients) or secondary bone grafting and plate stabilisation (2 patients after 12 weeks). Neither hardware failure nor wound complications were encountered.

## Discussion

Many operative techniques have been described for correction of hallux valgus. For severe deformities, proximal metatarsal osteotomies provide a high potential for correction because the corrective arc of rotation is greater compared to distal or shaft osteotomies [10]. In our study, we achieved significant correction of HVA and IMA with proximal chevron-like osteotomy combined with distal soft tissue procedure which is consistent with previous studies [7,8,11-13]. Comparing HVA and IMA 2 days and 6 weeks after surgery we found some loss of correction. This could be explained with an absence of full weightbearing due to pain shortly after surgery (for IMA) and the dressing bandage applied directly after surgery with maximal correction (for HVA). Even if patients continue the redressing bandages as suggested, a slight recurrence of hallux valgus (compared to the 2 day follow up) is often observed. In our experience, and with support from the literature, the majority of loss of correction (HV, IMA, first metatarsal length) occurs within the first 6 weeks after surgery [14]. The proximal chevron osteotomy is inherently more stable compared to osteotomies performed in one plane, but complications such as delayed union, non-union or malunion leading to first ray dorsiflexion and recurrence could occur for every type of osteotomy. In this context, the question of the best fixation method is still under discussion. Reviewing the literature there are several biomechanical studies on saw bones or cadaveric feet. Lian et al. and Bozkurt et al. reported screw fixation to be significantly stronger than K-wires or staples [15,16]. Fillinger et al. demonstrated a double screw fixation to be more stable compared to single screw fixation [17]. Varner et al. reported greater stability of plate fixation compared to single screw fixation for metatarsal crescentic osteotomy [18].

Dorsiflexion, malunion and shortening of the first metatarsal have been described as possible postoperative complications after proximal osteotomies of the first metatarsal, which can lead to transfer metatarsalgia [19-21]. The closing wedge osteotomy slightly shortens the first metatarsal, however Day et al. found that this does not significantly change clinical outcome and only shortens the first metatarsal slightly postoperatively [22]. The proximal chevron-like osteotomy incorporates the opening wedge principle and should lead to a slight lengthening. In our study, the mean length of the first metatarsal shortened by about 2.8 mm and mean first metatarsal protrusion decreased by 2.2 mm, both of which were not statistically significance (preoperative vs. 6 weeks postoperative). Variations between radiographic technique, degrees of magnification and level of weightbearing could have possibly affected the measurements. In addition, with our surgical technique the distal fragment is slightly plantarflexed relative to the proximal fragment to avoid postoperative dorsiflexion, which could lead to a supposed shortened first metatarsal in dorsoplantar X-rays. Another explanation discussed in the literature could be instability of the osteotomy leading to a prolonged bone healing with possible loss of metatarsal length and correction. For that reason, many authors prefer a plate for stabilisation of the osteotomy [4,6-8]. In addition, plate fixation is technically less challenging compared to single screw fixation [2,5,23]. Disadvantages of plate fixation compared to single screw fixation are higher costs, more metal load with possible hardware failure, higher chance of soft tissue irritation and larger wounds if plate removal becomes necessary.

Regarding postoperative treatment, most authors allow full weightbearing in a stiff-soled shoe or cast during the first two weeks after surgery, while some only allow heel weightbearing [4,7,8,11]. Early ambulation with full weightbearing reduces the risk of deep vein thrombosis and prevents muscular atrophy. The postoperative shoe or dressing is discontinued after six weeks. Our study confirms that single screw fixation of the proximal chevron osteotomy is stable enough to allow full weightbearing in a stiff-soled shoe from the first day after surgery. To protect the medial capsulorrhaphy we advise patients to apply dressing bandages, which are discontinued after six weeks.

Compared to attending surgeons, the mean operating time of residents was 20 minutes longer. During follow-up we found no significant differences between both groups concerning correction of HVA/IMA or postoperative complications. Nevertheless, the proximal chevron-like osteotomy is technically challenging and it should be noted that our operating residents are always assisted by an attending surgeon.

Our study has several limitations. We had only one surgeon performing all preoperative and postoperative measurements. As such, intraobserver errors cannot be excluded. We could not control variations between radiographic techniques, degrees of magnification and level of weightbearing of patients, especially 2 days after surgery. Finally, our follow-up was for only six weeks, although our study design allowed the relevant research questions to be adequately answered.

## Conclusions

The proximal chevron osteotomy with single screw stabilisation and full postoperative weightbearing using a stiff-soled shoe is a reliable and cost effective method for treating severe hallux valgus deformities with satisfactory results. Adequate surgical skills and high precision in screw placement are necessary.

## Competing interests

The authors declare that they have no competing interests.

## Authors’ contributions

FM: participated in collecting data and drafting manuscript. UL: participated in study design. CM: statistics. II: participated in collecting data. NW: participated in study design. MW: participated in study design, final approval. All authors read and approved the final manuscript.
